# The FANTASTIC FOUR proteins influence shoot meristem size in *Arabidopsis thaliana*

**DOI:** 10.1186/1471-2229-10-285

**Published:** 2010-12-22

**Authors:** Vanessa Wahl, Luise H Brand, Ya-Long Guo, Markus Schmid

**Affiliations:** 1 Max Planck Institute of Molecular Plant Physiology, D-14476 Potsdam, Germany; 2 Zentrum für Molekularbiologie der Pflanzen - Pflanzenphysiologie, Universität Tübingen, Auf der Morgenstelle 1, D-72076 Tübingen, Germany; 3 Department of Molecular Biology, AG Schmid, Max Planck Institute for Developmental Biology, D-72076 Tübingen, Germany

## Abstract

**Background:**

Throughout their lives plants produce new organs from groups of pluripotent cells called meristems, located at the tips of the shoot and the root. The size of the shoot meristem is tightly controlled by a feedback loop, which involves the homeodomain transcription factor WUSCHEL (WUS) and the CLAVATA (CLV) proteins. This regulatory circuit is further fine-tuned by morphogenic signals such as hormones and sugars.

**Results:**

Here we show that a family of four plant-specific proteins, encoded by the *FANTASTIC FOUR *(*FAF*) genes, has the potential to regulate shoot meristem size in *Arabidopsis thaliana*. *FAF2 *and *FAF4 *are expressed in the centre of the shoot meristem, overlapping with the site of *WUS *expression. Consistent with a regulatory interaction between the *FAF *gene family and *WUS*, our experiments indicate that the FAFs can repress *WUS*, which ultimately leads to an arrest of meristem activity in *FAF *overexpressing lines. The finding that meristematic expression of *FAF2 *and *FAF4 *is under negative control by CLV3 further supports the hypothesis that the FAFs are modulators of the genetic circuit that regulates the meristem.

**Conclusion:**

This study reports the initial characterization of the *Arabidopsis thaliana FAF *gene family. Our data indicate that the *FAF *genes form a plant specific gene family, the members of which have the potential to regulate the size of the shoot meristem by modulating the CLV3-WUS feedback loop.

## Background

In contrast to animals, plant development is highly plastic, with new organs being formed continuously from pools of stem cells maintained in structures called meristems. This plasticity allows plants, within certain limits, to adapt their body shape in response to developmental, physical and environmental cues. The ability to form new organs throughout their life cycle requires tight control of the meristems to avoid unregulated growth. Plants have evolved an elaborate genetic network that controls meristem size and maintenance [1,2]. At the core of the network that regulates the size of the stem cell population in the shoot meristem are the homeodomain transcription factor WUSCHEL (WUS) and the CLAVATA (CLV) ligand-receptor system [1,3-5]. *WUS *is expressed in the organizing centre (OC) of the meristem and positively regulates *CLV3 *expression in the stem cells, which are localized above the OC [6]. *CLV3 *encodes a small secreted peptide, which cell non-autonomously represses *WUS *in the OC [6-10]. It has recently been shown, that CLV3 directly binds to the ectodomain of the LRR receptor kinase CLV1 [11]. Similarly, it has been suggested that the receptor-like protein CLV2 interacts with the novel receptor kinase CORYNE (CRN; SUPPRESSOR OF OVEREXPRESSION OF LLP1-2, SOL2) to establish a functional CLV3 receptor [12,13]. Thus a feedback loop is established, which is essential to set up and maintain the stem cell population at the shoot meristem. However, the relationship between *WUS *and *CLV3 *is not static; the *WUS*-*CLV *system can compensate for changes in *CLV3 *expression over a wide range [14].

*WUS *expression is also controlled by phytohormones, which have been implicated in maintaining the stem cell system as well as setting up developmental compartments at the shoot meristem and in establishing the developmental fate of cells that are derived from the stem cell pool [reviewed in 2]. Besides hormones, sugars also appear to play an important role in establishing and maintaining meristem identity [reviewed in 15]. For example, it has been shown that growth arrest caused by loss of the *WUS*-related homeodomain factor *STIMPY*/*WOX9 *was rescued to a large extent by providing sucrose in the growth medium. This demonstrates that sucrose can compensate for the loss of at least some genes normally required for meristem development [16].

Here we present an initial characterization of a plant-specific gene family - *FANTASTIC FOUR *(*FAF*) - with four members in *Arabidopsis thaliana *(*FAF1 *- *FAF4*). We show that the *FAF *genes are expressed throughout the life cycle of the plant, but exhibit strong temporal and spatial regulation. *FAF2 *and *FAF4 *expression was detected in the centre of the shoot meristem by RNA *in situ *hybridization and GUS reporter constructs. In addition, expression of the *FAF *genes was detectable in the developing and mature vasculature. *FAF *gene overexpression negatively affected growth of both the shoot and the root. At the molecular level, the arrest of shoot growth was accompanied by a marked decrease in *WUS *expression. We further show that meristematic expression of *FAF2 *and *FAF4 *is under negative control by CLV3. Together these data suggest that the FAF proteins are capable of modulating shoot growth by repressing *WUS *in the OC of the shoot meristem.

## Results

### The *FANTASTIC FOUR (FAF) *genes define a plant specific gene family

The *Arabidopsis thaliana FAF *genes first caught our attention because two of them, *FAF1 *(At4g02810) and *FAF2 *(At1g03170), responded strongly and rapidly to a shift in photoperiod in a microarray experiment (Additional File 1 Figure S1) [17]. *FAF1 *and *FAF2 *belong to an uncharacterized gene family that also includes *FAF3 *and *FAF4 *(At5g19260, At3g06020, Table 1). Both pairs of genes, *Arabidopsis thaliana FAF1/FAF2 *and *FAF3/FAF4*, appear to be recently duplicated paralogs [18]. The proteins encoded by the *FAF *genes do not contain any domains of known function (Table 1). In addition, the *Arabidopsis thaliana *genome encodes a more distantly related protein (At5g22090), which we call *FAF-like *(Additional File 1 Table S1). FAF and FAF-like proteins share several conserved domains, among them a stretch of acidic residues in their C-terminal half.

**Table 1:** Properties of Arabidopsis thaliana FAF proteins

			Protein properties			
			
Gene	AGI	Annotation	Length (aa)	Mass (kDa)	pI	Domains of known function
*FAF1*	At4g02810	expressed protein	271	31.2	4.08	none
*FAF2*	At1g03170	expressed protein	240	27.3	4.74	none
*FAF3*	At5g19260	expressed protein	288	32.1	4.34	none
*FAF4*	At3g06020	expressed protein	300	33.9	4.88	none

Since the *FAF *genes have not been previously described, we wished to determine how widespread they are among other species. To address this question we searched publicly available sequence databases by reciprocal BLAST analysis for potential orthologs of the *FAF *genes. Phylogenetic analysis suggests that the *FAF *genes originated from a *FAF-like *gene and that today's *FAF *genes arose through several rounds of duplications within the dicotyledonous plants (Additional File 1 Figure S2). *FAF *genes were not apparent in the rice genome or any other monocotyledonous species, even though proteins sharing homology with the *Arabidopsis thaliana FAF-like *gene were clearly present (Additional File 1 Table S1). Sequence homology searches failed to identify any potentially homologous proteins outside the plant kingdom, indicating that the *FAF *gene family is plant-, possibly eudicotyledonous-specific.

### Expression of *FAF *genes throughout development

**Figure 1:**
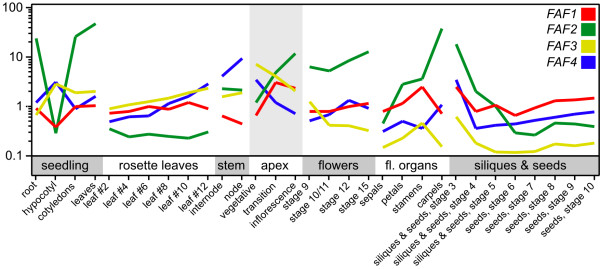
**Microarray expression profiles of the *FAF *gene family**. Expression of *FAF *genes in selected tissues from the 'AtGenExpress' expression atlas of *Arabidopsis thaliana *development. Samples confirming the expression changes observed at the apex during the floral transition (Additional File 1 Figure S1) are shaded.

In order to determine the temporal and spatial regulation of the expression of the four *FAF *genes throughout development, we consulted the AtGenExpress *Arabidopsis thaliana *expression atlas [19]. All four *FAF *transcripts were detectable throughout development (Figure 1). Expression of *FAF1 *and *FAF2 *at the shoot apex increased during the transition to flowering, while *FAF3 *and *FAF4 *decreased, confirming the results observed in the first microarray dataset (Additional File 1 Figure S1). However, *FAF1 *and *FAF2 *exhibited strong differences in their expression profiles in other tissues. For example, while *FAF1 *and *FAF2 *were both highly expressed in the apical region during the floral transition, only *FAF2 *expression was maintained at high levels during later stages of flower development, especially in carpels. In contrast, *FAF1 *expression appeared to be more transient, with some expression maintained in stamens. Similarly to *FAF2*, *FAF3 *was expressed in stamens, but was also strongly expressed in the youngest leaves formed by the plant (Figure 1). This expression, however, disappeared as the leaves aged. Expression of all four *FAF *genes was detectable in young siliques, but expression faded as seed maturation progressed. Taken together, our analysis of microarray data showed that the *FAF *genes are dynamically expressed throughout development.

### *FAF *genes are expressed in the centre of the shoot meristem and in vascular tissue

**Figure 2:**
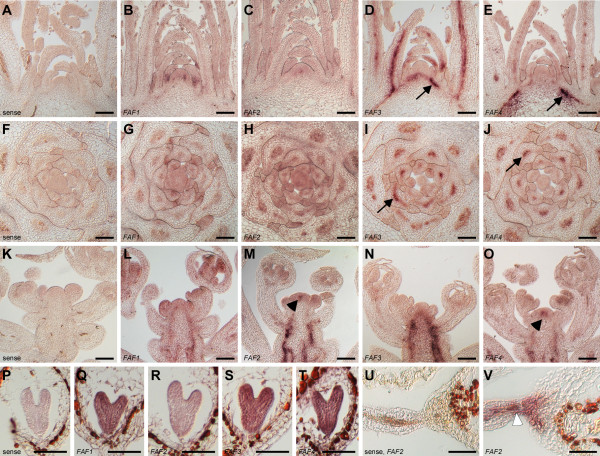
**Expression patterns of the *FAF *genes throughout development assayed by RNA *in situ *hybridization**. (**A**-**J**) Expression of the *FAF *genes at the vegetative apex. Longitudinal (**A**-**E**) and transverse sections (**F**-**J**) through the vegetative apex hybridized with sense (**A**, **F**) and antisense probes (**B**-**E**, **G**-**J**) against the four *FAF *genes are shown. Highest expression was detected for *FAF3 *and *FAF4 *in the vascular and provascular tissue (**D**, **E**, **I**, **J**, arrows). (**K**-**O**) In inflorescences, *FAF1 *expression (**L**) was detected in the developing vasculature and young flowers. *FAF2 *expression (**M**) was highest in the inflorescence stem, but also detectable in the centre of the meristem (**M**, arrowhead). Expression of *FAF3 *was restricted to the developing vasculature (**N**), while *FAF4 *was also found in the centre of the meristem (**O**, arrowhead). No signal was found when sense probes were used (**K**). (**P**-**V**) During embryogenesis, *FAF1 *(**Q**), *FAF3 *(**S**), and *FAF4 *(**T**) were expressed in the embryo from heart stage onward, while expression of *FAF2 *was limited to the funiculus (**V**). Sense probes (**P**, **U**) did not result in any staining. Scale bars: 100 μm (A-O), 50 μm (P-T).

To analyze *FAF *expression at cellular resolution, we carried out RNA *in situ *hybridization (Figure 2). Expression of all four *FAF *genes was detected in provascular and vascular tissue at different stages throughout development. *FAF1 *and *FAF*2 were only weakly expressed in the vasculature of vegetative plants (Figure 2B, C, G, H). In addition to the vasculature, *FAF2 *mRNA was also detectable in the centre of the vegetative meristem (Figure 2C). In contrast, *FAF3 *and *FAF4 *could easily be detected in the vasculature (Figure 2D, E, I, J; arrows), but neither seemed to be expressed in the vegetative meristem (Figure 2D, E).

Expression of the *FAF *genes changed upon the onset of flowering (Figure 2L-O), as already observed in the microarray experiments (Figure 1). *FAF1 *and *FAF*2 were induced in the inflorescence vasculature and young flower buds as flowering commenced (Figure 2L, M). In contrast, *FAF3 *and *FAF4 *expression in inflorescences was restricted to the vasculature, but was largely absent from young flowers (Figure 2N, O). Both, *FAF2 *and *FAF4 *were, however, detected in the centre of the inflorescence meristem (Figure 2 M, O; arrowheads).

Upon fertilization, expression of *FAF1, FAF3, FAF4*, but not *FAF2 *could also be detected in the developing embryo, starting from the early heart stage and lasting until torpedo stage (Figure 2Q-T). *FAF2 *expression was, however, detectable in the funiculus (Figure 2V, arrowhead).

The dynamic nature of *FAF *gene regulation was confirmed by the dramatic changes in reporter gene activity observed during the first 8 days after germination (Additional File 1 Figure S3). *FAF1::GUS *activity, for example, was initially restricted to the hypocotyl, but expression gradually shifted to the root over the following four days. Starting on day 6, *FAF1::GUS *became active in the vasculature of the cotyledons and subsequently also in the leaves. Similar, but distinct, dynamic regulation of reporter gene activity could also be observed for the other *FAF *promoters (Additional File 1 Figure S3). In addition, *FAF2::GUS *was observed in the centre of the vegetative shoot meristem (Additional File 1 Figure S4H) as already shown by RNA *in situ *hybridization (Figure 2M). After the onset of flowering, *FAF1::GUS *was observed most strongly in anthers (Additional File 1 Figure S4A), while *FAF2::GUS *expression was strongest in the carpel, particularly in the funiculus (Additional File 1 Figure S4B, G), where *FAF2 *RNA had also been detected (Figure 2V). *FAF3::GUS *activity was restricted to anthers (Additional File 1 Figure S4C), whereas *FAF4 *was expressed at the base of the flower and in the vasculature of the pedicels and the inflorescence stem (Additional File 1 Figure S4D). In differentiated tissues such as root and leaves, the *FAF *genes were predominantly expressed in the phloem, as shown for *FAF2 *(Additional File 1 Figure S3E, F).

In summary, *FAF2 *and *FAF4 *are expressed in the centre of the shoot meristem, suggesting a potential role for these two FAF proteins in meristem development. In addition, all *FAF *genes are expressed in the vasculature, where they may function in a partially redundant manner.

### FAF proteins can affect growth and meristem size

To study the function of the FAF proteins during development, we first searched for knock-out lines (Additional File 1 Table S2). Most of the lines investigated showed either wild-type mRNA levels, indicating that expression of the corresponding *FAF *gene was unaltered in these lines or the presence of the T-DNA could not be confirmed or the lines were not available from the stock centre. Only for *FAF3 *a potential RNA-null line (SM_3_40331) could be recovered. This line, however, did not show an obvious phenotype, possibly due to redundancy with the other *FAF *genes. Attempts to knock-down individual or certain combinations of *FAF *genes by constitutive and inducible RNAi (Additional File 1 Table S3) resulted in pleiotropic phenotypes in all T1 lines investigated. Unfortunately, all lines that eventually did set seeds were silenced in T2, making further analysis impracticable. Besides regular RNAi, artificial microRNAs (Additional File 1 Table S4) were prepared to knock-down *FAF *mRNAs either individually or in combination, but these did not result in a significant degradation of the targeted transcripts and lines showed no discernable phenotypes [20,21]. Finally, tilling of *FAF *genes (Additional File 1 Table S5) also failed to produce alleles with major changes such as premature stop codons [22,23].

**Figure 3:**
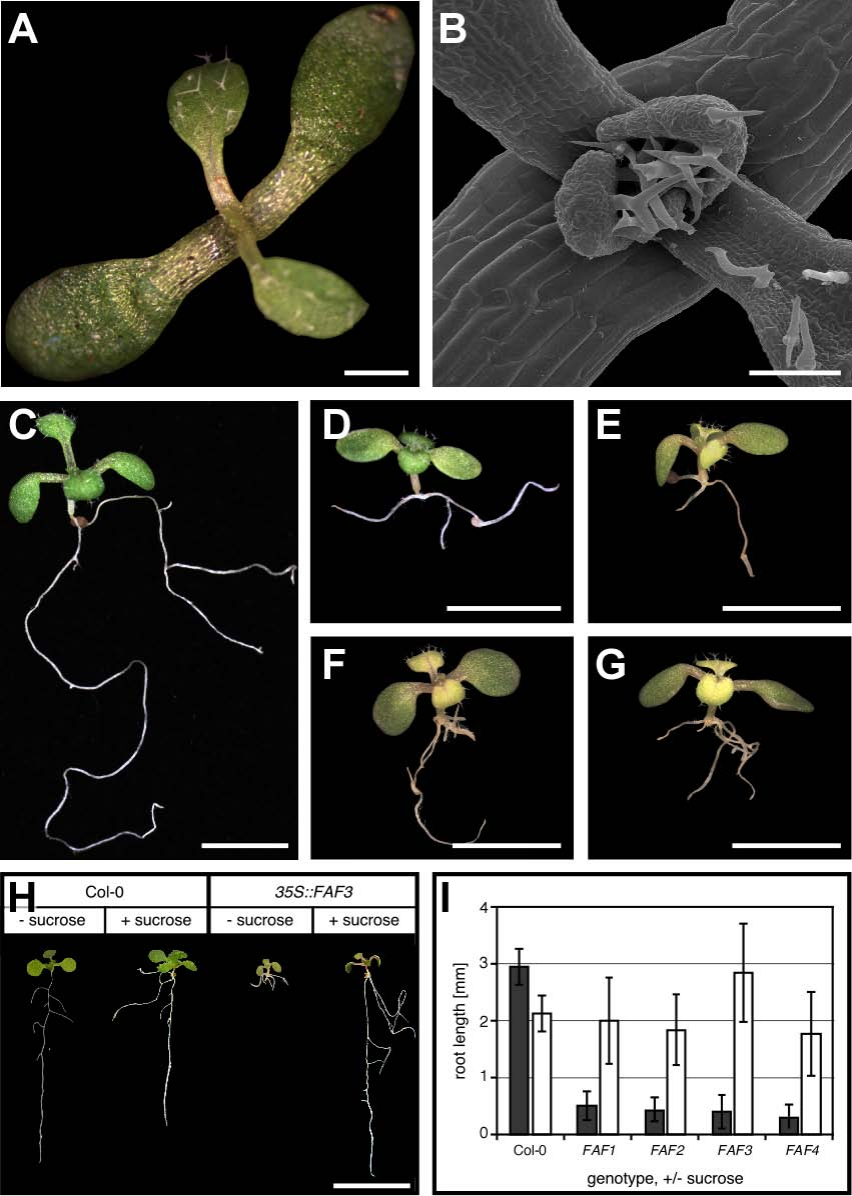
**Arrest of shoot and root growth by constitutive *FAF *expression**. (**A**) Arrested shoot meristem in a strong *35S::FAF3 *seedling. Expression of the other *FAF *genes by the 35 S promoter caused similar phenotypes (data not shown) (**B**) Close-up of arrested seedling under the SEM. (**C-G**) Root development of wild-type control (**C**) and intermediate *35S::FAF1 *(**D**), *35S::FAF2 *(**E**), *35S::FAF3 *(**F**), and *35S::FAF4 *(**G**) plants. The growth of the primary root is inhibited and the formation of adventitious roots is induced by high levels of *FAF *expression (**D-G**). (**H**) Rescue of root growth of a *35S::FAF3 *line by exogenous sucrose (1%). (**I**) Quantification of the effect of sucrose on root growth in Col-0 and *35S::FAF *plants (n = 20). Scale bars: 0.5 mm (A), 200 μm (B), 5 mm (C), 2 mm (D-G), 1 cm. (H).

Given the difficulty of obtaining loss-of-function lines, we resorted to misexpression experiments. We constitutively expressed *FAF *genes under the control of the viral *35 S *promoter *in planta*. In general we observed similar phenotypes, regardless of which *FAF *gene was overexpressed, indicating that all four FAF proteins can perform the same function. Lines expressing *FAF *genes at a very high level, as determined by qRT-PCR (data not shown), arrested shoot growth shortly after germination (Figure 3A, B). Arrest this early in development was observed in 2% (*FAF2*) to 12% (*FAF3*) of independent T1 lines (n > 140 per *FAF *gene).

The strongest lines were sterile, therefore we focused our analysis on those plants with intermediate expression levels (21% to 36% of independent T1 lines), for which stable lines could be established. In these lines we observed a strong reduction in root growth (Figure 3D-G) when compared to wild-type plants (Figure 3C). This was accompanied by an increased formation of adventitious roots at the hypocotyl. The arrest of the root growth could be overcome when 1% sucrose was supplied in the medium (Figure 3 H, I).

**Figure 4:**
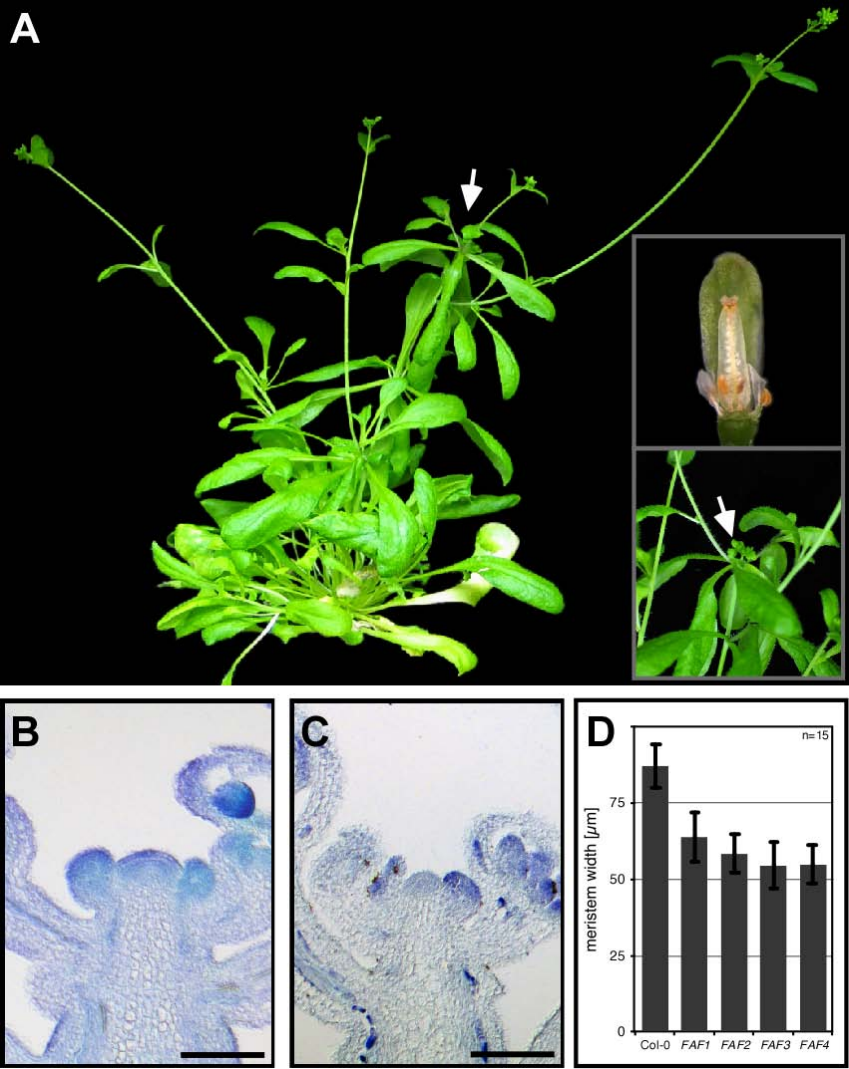
**Arrest of inflorescence and floral meristem by constitutive *FAF *expression**. (**A**) Phenotype of an intermediate *35S::FAF3 *plant. The inflorescence meristem of the main shoot has arrested growth (arrow and lower inset). Flowers derived from arrested meristems also display a growth arrest phenotype (upper inset). (**B **and **C**) Longitudinal section through wild-type (**B**) and *35S::FAF3 *inflorescences (**C**) stained with toluidine blue. (**D**) Quantification of inflorescence meristem width in control and *35S::FAF *plants. Meristem width is reduced in all four *FAF *overexpressing lines by approximately 30%. Scale bar: 100 μm; error bars: standard deviation (SD), n≥15.

Moderate *FAF *overexpressing plants were smaller than wild-type, and leaf vasculature appeared to be reduced (not shown). Apart from this they developed normally, until after the transition to flowering and bolting, at which point inflorescence meristems ceased producing new organs and shoot elongation stopped (Figure 4A and inset). In the last flowers to be formed before the meristem arrested, floral organs, in particular the stamens and carpel, were retarded in development (Figure 4A, inset). When we examined the meristems in more detail (Figure 4B, C), we found that the width of the inflorescence meristems in *FAF *overexpressing lines was on average reduced by approximately 30% when compared to wild-type (Figure 4D).

### FAF proteins can repress *WUSCHEL *in the organizing centre of the shoot meristem

**Figure 5:**
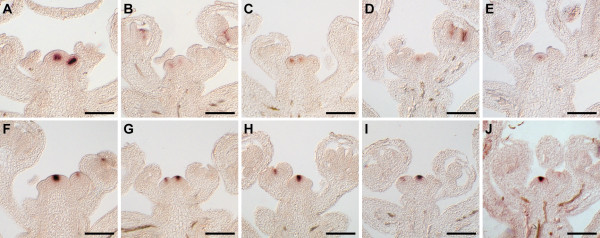
**Effect of *FAF *genes on *WUS *and *CLV3 *gene expression**. Detection of *WUS *(**A-E**) and *CLV3 *(**F-J**) transcripts by RNA *in situ *hybridization in wild-type (**A, F**), *35S::FAF1 *(**B, G**), *35S::FAF2 *(**C, H**), *35S::FAF3 *(**D, I**), and *35S::FAF4 *(**E, J**). *WUS *expression is reduced (**B-E**) while *CLV3 *expression (**G-J**) appears normal in *35S::FAF *plants. Scale bar: 100 μm.

Loss of *WUS *function results in a reduction of meristem size, similar to what we observed in *FAF *overexpressing lines. Moreover, two *FAF *genes are expressed in the centre of the meristem, overlapping with the site of *WUS *expression in the OC. This prompted us to analyze expression of *WUS *in the meristem of *FAF *overexpressing lines (Figure 5A-E). We found that *WUS *expression was strongly reduced in both inflorescence and flower meristems. Since WUS is required for maintenance of meristem function, the reduction in *WUS *expression is consistent with the meristem arrest phenotype seen in strong (Figure 3A) and moderate (Figure 4) *FAF *overexpressing plants.

Expression of *WUS *in the OC of the shoot meristem is under negative control of CLV3-dependent signalling. We found that *CLV3 *expression was essentially normal in *FAF *overexpressing lines (Figure 5F-J), indicating that the reduction in *WUS *expression was not caused by an increase or expansion of *CLV3 *expression.

### Repression of *FAF2 *and *FAF4 *in the shoot meristem by CLAVATA3

**Figure 6:**
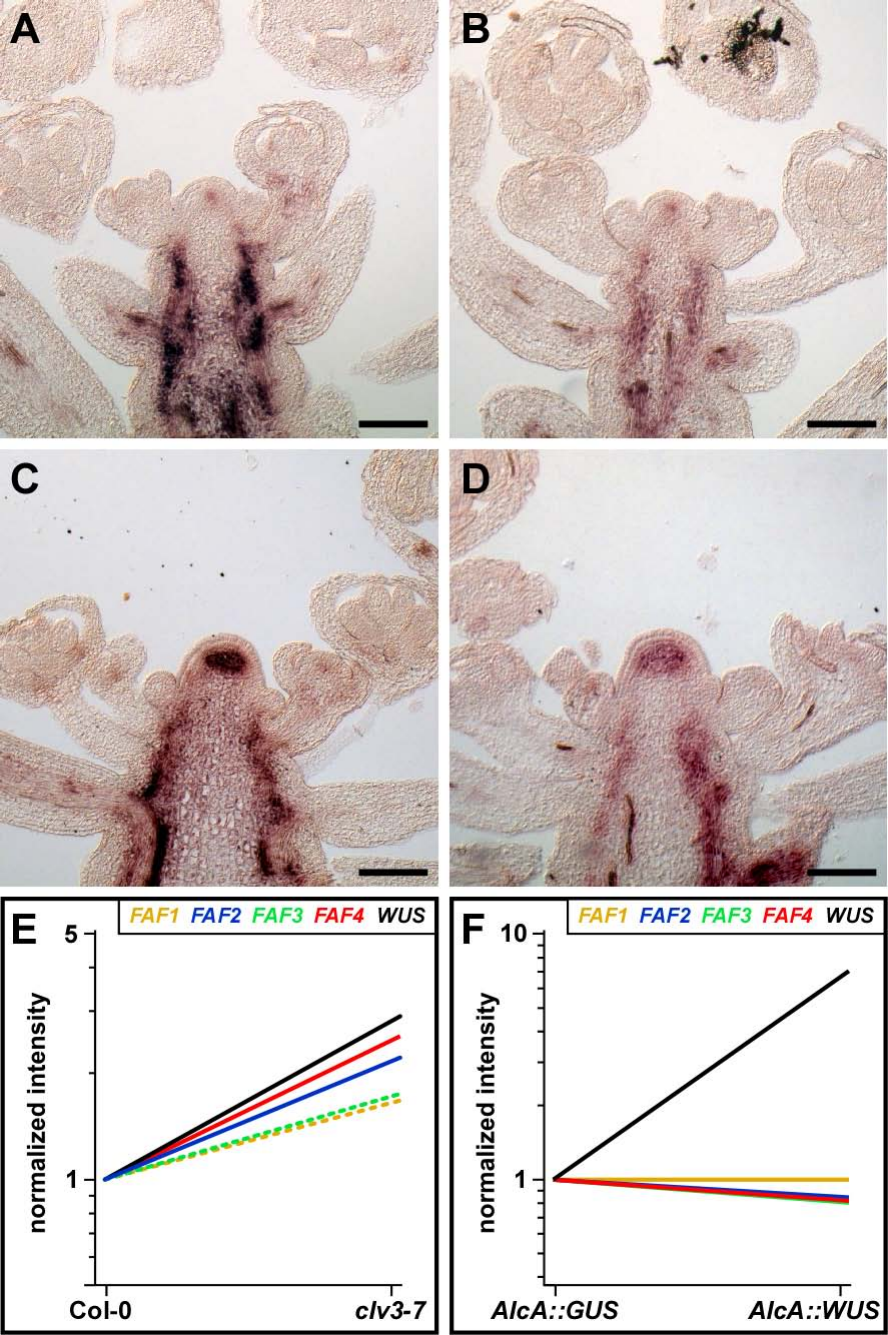
**Negative regulation of *FAF2 *and *FAF4 *expression in the organizing centre of the shoot meristem by CLV3**. Expression of *FAF2 *(**A, C**) and *FAF4 *(**B, D**) in wild-type control plants (**A, B**) and *clv3-7 *mutants (**C, D**). Expression of *FAF2 *(**C**) and *FAF4 *(**D**) is elevated in *clv3-7 *mutants when compared to wild-type controls (**A, B**). (**E, F**) Microarray expression profiles of *WUS *and the *FAF *genes. (**E**) *WUS*, *FAF2*, and *FAF4 *are significantly upregulated and change more than 2-fold (solid lines) in *clv3-7*, while *FAF1 *and *FAF3 *do not (dashed lines). (**F**) *FAF *genes do not respond to ectopic *WUS *expression. Scale bar: 100 μm.

The fact that *WUS *expression is reduced in *FAF *overexpressing lines suggested that *FAF2 *and *FAF4*, which are normally expressed in the meristem, might be involved in the CLV3 mediated repression of *WUS*. We therefore analyzed *FAF2 *and *FAF4 *expression in *clv3-7 *mutants (Figure 6). We found that expression of *FAF2 *was strongly enhanced in the centre of *clv3-7 *inflorescence meristems (Figure 6A, C), while its expression in the vasculature appeared to be not affected. Although meristems are enlarged in *clv3-7 *mutants, the simple increase in cell number does not explain the strong staining observed, suggesting that *FAF2 *is under repression by CLV3. Similarly, we found *FAF4 *to be expressed more strongly in the enlarged centre of *clv3-7 *meristems (Figure 6B, D), though the increase was not as pronounced as for *FAF2*. In order to confirm the upregulation of *FAF2 *and *FAF4 *in the inflorescence meristem of *clv3-7 *mutants, we analyzed microarray expression data of Col-0 and *clv3-7 *inflorescence meristems from the AtGenExpress transcriptome atlas. We found significant (logitT p < 0.01) and strong induction of *FAF2 *(2.2-fold) and *FAF4 *(2.5-fold) in *clv3-7 *inflorescence meristems when compared to Col-0 control plants (Figure 6E). Confirming the quality of the array data, *WUS *was also found to be significantly and strongly (2.9-fold) induced in the *clv3-7 *mutant. Neither *FAF1 *nor *FAF3 *changed significantly and strongly (> 2-fold) in the *clv3-7 *microarray data set.

The observed upregulation of *FAF2 *and *FAF4 *in *clv3-7 *inflorescence meristems could either indicate that these two *FAF *genes are under repression by CLV3 or that they are positively regulated by WUS. To be able to distinguish between these two possibilities we examined the response of *FAF *genes to inducible ectopic *WUS *expression in a microarray dataset from 12-day-old seedlings [24]. We found that none of the *FAF *genes were induced, suggesting that they are not positively regulated by WUS but are more likely to be under repression by CLV3 (Figure 6F).

Taken together, our results indicate that FAF proteins, when expressed at high levels, can affect shoot meristem size in *Arabidopsis thaliana *by modulating CLV3-dependent *WUS *expression. In wild-type plants, only *FAF2 *and *FAF4 *are likely to participate in the regulation of *WUS *since only these two genes are normally expressed in the centre of the shoot meristem. In addition, *FAF2 *and *FAF4 *expression in the meristem appears to be under negative control by the CLV3. However, the observation that constitutive expression of any of the four *FAFs *can affect meristem size demonstrates that the ability to repress *WUS *is intrinsic to all four FAF proteins.

## Discussion

The shoot apical meristem is initiated early during embryogenesis and harbours a small population of pluripotent stem cells from which all aerial parts of the plant are derived [1,25]. Establishment and maintenance of these stem cells depends on the activity of the *WUS *and *CLV *genes, which are mutually regulating each other's expression in a spatial negative feedback loop [3]. *WUS *expression in the OC of the shoot meristem promotes stem cell fate in the cells above while the stem cells themselves secrete a small peptide, CLV3, which is perceived by CLV1 and, possibly, the CLV2/CRN receptor complex [3,11,12,26]. Ultimately, CLV3-dependent signalling limits the size of the *WUS*-expressing OC. The WUS-CLV system is rather dynamic and can, over time, compensate for even 10-fold differences in *CLV3 *expression, indicating that *CLV3 *expression confers information about stem-cell position to the underlying OC rather than information about stem cell number [14].

Analysis of *FAF *overexpressing lines by RNA *in situ *hybridization demonstrated that *WUS *was strongly downregulated in these lines. The fact that the expression of *WUS *was affected regardless of which *FAF *gene was constitutively expressed, suggests that the ability to repress *WUS *is intrinsic to all four FAF proteins. In wild-type, *FAF *effects on *WUS *are likely to be exerted only by *FAF2 *and *FAF4*, which are the two *FAF *genes expressed in the centre of the shoot and/or inflorescence meristem in a domain that appears to be overlapping with the site of *WUS *expression.

In the *clv3-7 *mutant the expression domains of *WUS *and *FAF2*/*FAF4 *appear to be largely exclusive. *WUS *is limited to the second meristem layer (L2) but is no longer detectable in the centre of the meristem [7,27]. In contrast, expression of *FAF2 *and *FAF4 *were found to be upregulated in the centre of the meristem but are mostly excluded from the L2. This suggests that in wild-type expression of *FAF2*/*FAF4 *might attenuate *WUS *expression in the centre of the meristem whereas high levels of *FAF2*/*FAF4 *in *clv3-7 *prevent *WUS *from being expressed in the centre of the meristem and limit its expression to the L2. Based on our results, we propose that *FAF *genes function in the shoot meristem, with CLV3 negatively regulating *FAF2 *and *FAF4 *expression, which in turn contribute to the repression of *WUS*. In this context it is interesting to note that all four FAF proteins harbour a short sequence motif (L-X-L-X-L) that is reminiscent of the EAR repression motif [28]. This would be in agreement with the proposed role of FAF proteins as repressors of *WUS*.

Expression of *FAF2 *and *FAF4 *in the centre of the meristem would put them in place to compensate for the effects of positive regulators such as STIMPY on *WUS *expression in the OC. Interestingly, we found that *CLV3 *expression was not decreased in *FAF *overexpression lines, even though *WUS *levels were severely reduced. Expression of *WUS *in the OC is under constant surveillance by several other positive and negative regulators [reviewed in 1, 29]. For example, in *jba-1 D *plants, a mutant in which the *miR166g *is overexpressed, *WUS *expression is highly induced, while the relative level of *CLV3 *transcription remains unchanged compared with wild-type plants [30]. These observations together with data presented here suggest that the expression of *CLV3 *is maintained over a wide range of *WUS *levels, similar to what has been shown for the effect of CLV3 on *WUS *[14]. In addition, several other transcription factors, as well as a number of proteins involved in chromatin remodelling, have been shown to regulate *WUS*. Having established the FAF proteins as negative regulators of *WUS*, it will be interesting to analyze possible genetic interactions between the *FAF *genes and the other *WUS *regulators in detail.

*WUS *is not only expressed in the OC of the shoot meristem, but also in young flower meristems, where it directly regulates expression of the homeotic gene *AGAMOUS *(*AG*) in the centre of the newly formed flower [31,32]. AG is normally required for the development of the inner two whorls of the flower [33]. Reduction of *WUS *expression in the flower meristem could result in a downregulation of *AG*, which could explain the observed defects in flowers of *FAF *overexpressing plants.

Apart from defects in the shoot meristem, *FAF *overexpression resulted in an arrested root meristem. This finding suggests that the FAF proteins can influence meristem maintenance at both poles of the growing plant. Since *WUS *is not expressed in the root meristem, it will be interesting to investigate, which *WOX *gene takes on its function in the root. STIMPY (STIP; WOX9), a homeodomain transcription factor related to WUS, has recently been shown to promote *WUS *expression in the vegetative shoot meristem [16]. Based on the severity of loss-of-function alleles on both the shoot and the root meristems, STIP seems to play a more general role in meristem maintenance than WUS. In this context it is interesting to note that, similar to *FAF *overexpression, loss of *STIP *function can be compensated for by exogenous sucrose, which is in agreement with the proposed function for STIP in maintaining cell division. This suggests that STIP and the FAFs might have opposing functions in integrating sugar signalling into the meristem maintenance network.

The FAF proteins are likely to have functions other than meristem maintenance since all are expressed in vascular tissue. Consistent with a functional role for the FAFs in these tissues, we observed a reduction of tertiary and quaternary vein formation in *FAF *overexpressing lines (data not shown). It has been reported that *CLV1 *and a *CLV1-like *gene are expressed in the phloem and cambium. Also, two members of the *CLAVATA3/ESR-RELATED *(*CLE) *family, *CLE6 *and *CLE26*, are preferentially expressed in the phloem and/or the cambium [34], and it has recently been shown that application of dodecapeptides with two hydroxyproline residues encoded by the *CLE *gene family suppress xylem cell differentiation and promote cell division in *Zinnia *cell cultures [35]. Thus it seems possible that FAFs affect vascular development by a mechanism similar to the one we propose for FAF function in the shoot meristem. In such a scenario the FAF proteins would act as general repressors of cell division in both the cambium and the root and shoot meristem, but are themselves under the control of the different CLAVATA/CLE proteins. Taken together our findings suggest that FAF proteins might act as transcriptional regulators, the question how exactly they exert their function remains to be determined.

## Conclusions

Our study demonstrates that the four *Arabidopsis thaliana FAF *genes most likely arose from the *FAF-like *gene present in both monocotyledonous and dicotyledonous plant species, through two rounds of gene duplications. The expression of the *FAF *genes is under developmental regulation and individual *FAF *genes are expressed in distinct, though overlapping domains. The latter suggests that the FAF proteins might act partially redundant, which would explain why T-DNA insertion lines (as far as they could be confirmed) were indistinguishable from wild-type plants. Consistent with a certain amount of redundancy among the *FAF *genes, RNAi and artificial microRNAs to knock-down individual or at maximum two *FAF *genes also did not result in any consistent and reproducible phenotypes. Based on the expression of *FAF2 *and *FAF4 *in the centre of the shoot apex, however, we assume a role of these two members of the FAF family in the shoot meristem. Supporting this idea was the finding that constitutive overexpression of the *FAF *genes resulted in a marked reduction of meristem size. In addition, expression of *WUS*, a central player in the regulation of meristem size was strongly reduced in the *FAF *misexpression lines. Finally, expression of *FAF2 *and *FAF4 *themselves appear to be under the control of the WUS-CLV3 feedback loop, as these two *FAF *genes were strongly induced in the meristem of a *clv3 *mutant. Taken together, our data suggest a scenario in which FAF2 and FAF4 modulate meristem size while the function of the other two *FAF *genes remains to be investigated.

## Methods

### Plant material

All lines analyzed were in the Columbia (Col-0) background. Plants were grown either under long day (LD, 16 h light, 8 h darkness) or short day (SD, 8 h light, 16 h darkness) conditions at 65% relative humidity under a 2:1 mixture of Cool White (Sylvania, #0001510) and Warm White (Sylvania, #0001511) fluorescent lights, with a fluence rate of 125 to 175 μmol m^-2^s^-1^.

### Phylogenetic analysis

Potential homologs of the *Arabidopsis thaliana *FAF and FAF-like proteins were identified by reciprocal BLAST analysis. First, we queried public databases (NCBI; Phytozome V4) using 'tblastn' and 'blastp' (E < 1e-5) to identify potentially homologous proteins. Second, all candidates were checked against TAIR 9 protein database by 'blastp'. For this either the full length proteins (when available) or the longest peptides encoded by the various ESTs were used. Only proteins that resulted in an *Arabidopsis thaliana *FAF or the FAF-like protein as best hit were considered to be true FAF orthologs. For phylogenetic analysis, FAF and FAF-like proteins were preselected for maximum diversity. In particular, redundant sequences from the same or closely related species were not considered and only one representative protein sequence was included in the final tree. Peptides deduced from ESTs were only considered if they completely covered the conserved domains that were eventually used to construct the phylogeny. The only exception to this was a sequence originating from *Selaginella moellendorffii *(Phytozome-Id: 418746) that serves as an outgroup, which contains only one of the two regions that are conserved in all FAF and FAF-like proteins. Finally, the homologs of FAF proteins were aligned with T-COFFEE [36], then only the conserved domains were used for phylogenetic analysis. PAUP* version 4.0b10 [37] was used to reconstruct the phylogenetic tree using the Neighbor-joining (NJ) method. Topological robustness was assessed by bootstrap analysis with 1000 replicates using simple taxon addition [38].

### Analysis of microarray expression data

Microarray data were imported into the GeneSpring 7 software (Agilent Technologies) and normalized using gcRMA, implemented in GeneSpring 7 [39]. Additional 'per gene' normalization was performed in GeneSpring 7. Significant changes in gene expression were calculated using logit-T with a cut-off of p < 0.025 [40]. Lists of differentially expressed genes were imported into GeneSpring 7 for further analysis.

### Molecular work and cloning

All constructs created in this study that involved PCR were confirmed by DNA sequencing. See Additional File 1 Table S6 for information on the sequences of the oligonucleotides used. All four *FAF *genes are encoded by single exon genes. For the construction of overexpressing lines, protein coding region were amplified from genomic DNA and cloned into the pCRsmart vector, a derivative of pBluescript. ORFs were than cloned as *Bam*HI-*Pst*I fragments into the shuttle vector pBJ36-35 S. Cassettes containing the *35 S *promoter, the *FAF *ORF and the *ocs *terminator were excised from the respective plasmids using *Not*I, ligated into the pMLBART binary vector and transformed into Col-0 wild-type plants by floral dipping [41]. For the β-glucuronidase (GUS) reporters, 2.5 kb fragments upstream of the *FAF *start codon were amplified by PCR, cloned into the vector pRITA, which contains the *GUS *gene followed by a *nos *terminator. The entire cassettes were excised with *Not*I and ligated into the pMLBART binary vector that provides resistance to the herbicide glufosinate (Basta, Bayer CropScience) in plants.

### Scanning electron microscopy (SEM)

Tissue was fixed for 5 minutes in 100% methanol, followed by 3-5 washes with 100% ethanol. Further preparation was carried out as described [42]. Images were acquired on a Hitachi S800 electron microscope, at an accelerating voltage of 20 kV.

### RNA *in situ *hybridization and GUS staining

RNA *in situ *hybridization was performed largely as previously described [42], but infiltration with paraffin was carried out using an ASP300 automated embedding apparatus (Leica). Sections (9-12 μm) were prepared with an EG1160 microtome (Leica). Sense probes were tested for all genes, but did not result in any noticeable staining and were therefore omitted from most figures. Sections shown in different panels in a given figure were processed in parallel and the signal was allowed to develop for the same time to ensure comparability. Images were taken on an Axioplan2 microscope (Zeiss) equipped with an AxioCam HRc (Zeiss) digital camera. GUS staining was carried out as described [42]. Whole mount preparations were examined under an MZ FLIII (Leica) microscope and pictures were taken with an AxioCam HRc digital camera (Zeiss). Thin sections of tissues stained for GUS activity were prepared from paraffin embedded tissue as described above.

The width of the inflorescence meristem was determined on tissue sections stained with toluidine blue. For this purpose, serial sections of the meristem were prepared and the width of the meristem was determined from the section that passed through the centre of the meristem. The average meristem width and the standard deviation were calculated based on measurements of 15 meristems.

## Authors' contributions

VW and MS conceived and designed the experiments. VW performed all the experiments, except for some *in situ *hybridizations and the phylogenetic analysis, which were carried out by LHB and YG, respectively. VW and MS analyzed the data. VW and MS wrote the paper. All authors read and approved the final manuscript.

## Supplementary Material

Additional file 1 **• Table S1**. FAF-like proteins from *Arabidopsis thaliana *and several monocotyledonous species. • Table S2. *FAF *T-DNA insertion lines in Col-0 background. • Table S3. *FAF *RNAi hair-pin constructs. • Table S4. Artificial miRNAs targeting *FAF *transcripts. • Table S5. Summary of *FAF *tilling lines. • Table S6. Oligonucleotides used in this study. • Figure S1. Expression profiles of *FAF *genes in response to long day. • Figure S2. Phylogenetic analysis of the plant-specific *FAF *protein family. • Figure S3. *GUS *expression in seedlings of *FAF *reporter lines. • Figure S4. GUS reporter activity in the meristem and reproductive organs. Click here for file
