# Supplementary material for: The FANTASTIC FOUR proteins influence shoot meristem size in Arabidopsis thaliana
Source: BMC Plant Biol. 2010 Dec 22;10:285. doi: 10.1186/1471-2229-10-285 (PMC3023791; doi:10.1186/1471-2229-10-285)
Supplement: Additional file 1 — • Table S1. FAF-like proteins from Arabidopsis thaliana and several monocotyledonous species. • Table S2. FAF T-DNA insertion lines in Col-0 background. • Table S3. FAF RNAi hair-pin constructs. • Table S4. Artificial miRNAs targeting FAF transcripts. • Table S5. Summary of FAF tilling lines. • Table S6. Oligonucleotides used in this study. • Figure S1. Expression profiles of FAF genes in response to long day. • Figure S2. Phylogenetic analysis of the plant-specific FAF protein family. • Figure S3. GUS expression in seedlings of FAF reporter lines. • Figure S4. GUS reporter activity in the meristem and reproductive organs. [file 1471-2229-10-285-S1.PDF]

SUPPORTING INFORMATION

The FANTASTIC FOUR proteins influence shoot meristem size in *Arabidopsis thaliana*

Vanessa Wahl, Luise H. Brand, Ya-Long Guo, Markus Schmid

Table S1: Properties of FAF-like proteins from *Arabidopsis thaliana* and several monocotyledonous species.

		Protein properties			
**Description** [1]	**Annotation**	**Length (aa)**	**Mass (kDa)**	**pI**	**Domains of known function**
At5g22090	expressed protein	463	51.4	4.57	none
Oryza sativa / XP_469461	hypothetical protein	424	45.4	4.93	none
Sorghum bicolor / AAL75990	putative protein	438	47.7	5.27	none
Zea mays / AAP94588	putative protein	423	46.1	5.42	none
Zea mays / AAL76003	hypothetical protein	525	57.3	5.53	none

(1) AGI code is given for *Arabidopsis* protein (top), NCBI identifier for FAF-like proteins from other species (bottom).

Table S2: *Arabidopsis thaliana FAF* T-DNA insertion lines.

Gene	AGI	T-DNA Line	insertion in	RNA levels	comment
FAF1	At4g02810	GABI_454B12	5′ UTR	wt	
		GABI_339B07	3′ UTR	wt	
		WiscDsLox359E10	exon		1
		SK18570	exon		2
FAF2	At1g03170	SALK_131586.35.05.x	5′ UTR	up	
		SALK_105146.25.30.x	5′ UTR		3
		SALK_089736.19.90.x	exon		1
FAF3	At5g19260	GABI_299G01	5′ UTR	wt	
		SM_3_40331	exon	down	
FAF4	At3g06020	SM_3_41463	exon		4

1) T-DNA insertion could not be confirmed; 2) activation tagging line, not tested; 3) not tested; 4) not available from stock centre.

Table S3: *Arabidopsis thaliana FAF* RNAi hair-pin constructs.

Template	Primer	Sequence[1]	Region[2]	Target[3]
At4g02810	G-0757	gggtctagactcgagGGAGGGCATTGTGGGGAATAATG	666 - 816	*FAF1*
	G-0758	gggggatccgaattcTTAAGTGGTCACCCAAAATTGCTG		
At1g03170	G-0759	gggtctagactcgagGGAGGGCATCGACGAGGAAACTAG	579 - 729	*FAF2*
	G-0760	gggggatccgaattcTTAAGTAGCGACCCAAAACTGCTG		
At5g19260	G-0765	gggtctagactcgagGAGGAGGAGGAGGAAGAGGAAG	727 - 873	*FAF3*
	G-0766	gggggatccgaattcTTAGGAAGTAGCAACGCAGAAAG		
At3g06020	G-0767	gggtctagactcgagGAATACAAGGAAGAAGAAGAAG	766 - 912	*FAF4*
	G-0768	gggggatccgaattcTTAGGAAGTGGCAACACATAAAG		
At4g02810	G-0761	gggtctagactcgagATGTCCATCGTTGTTGGCCAAGC	1 - 148	*FAF1*
	G-0762	gggggatccgaattcAAGCTGTTTTCTTTGTGTCGTC		*FAF2*
At1g03170	G-0763	gggtctagactcgagATGTCACTTGTTGTTTGTCAAC	1 - 136	*FAF2*
	G-0764	gggggatccgaattcAAGCTTTATCCTCTTTGGTTTG		*FAF1*
At5g19260	G-0769	gggtctagagaattcCAAATCCTCACTTTTCTCAACC	86 - 275	*FAF3*
	G-0770	gggggatccggtaccCTTGTCGTCGTCTTTGTGGAG		*FAF4*
At3g06020	G-0771	gggtctagagaattcCAAATCCTCAACTATCCACACC	92 - 288	*FAF4*
	G-0772	gggggatccggtaccCTTCTCTTTATCATTAGAGCTC		*FAF3*

1) Forward primers introduce XbaI (tctaga) and XhoI (ctcgag) restriction sites, reverse primers introduce BamHI (ggatcc) and EcoRI (gaattc) restriction sites for cloning of hair-pin constructs into the pHANNIBAL vector; all RNAi constructs were expressed under the control of the 35S and AlcA promoter; 2) indicates positions of the first and last base of the fragment of a particular *FAF* ORF used to construct the RNAi hair-pin; 3) predicted targets of a particular *FAF* RNAi construct.

Table S4: Artificial miRNAs targeting *Arabidopsis thaliana FAF* transcripts.

Target[1]	Name	amiRNA[2]	Off targets	Promoters[3]
FAF1	amiRFAF1-I	TCCTTCGGGACGTTCAGACAC	At3g10610	35S
	amiRFAF1-II	TACGTTCGGATACAAAGCCGC		35S
FAF2	amiRFAF2-I	TAAGTAGGGACCCAAAGCCGC		35S
	amiRFAF2-II	TTTCAACGAACTTTATCGGGG	At2g05830	35S, XVE, HSP
	amiRFAF2-III	TTGAACTACAATTCGGCCGTC		35S, XVE, HSP
FAF3	amiRFAF3-I	TAAAGCCGTAGGCTCACTCAG	At3g60390	35S
FAF4	amiRFAF4-I	TCATGAAGGATATCGGGGCCC	At2g13560	35S
	amiRFAF4-II	TAATCAATCAAGATAGCGGGT		35S, XVE, HSP
	amiRFAF4-III	TCAACGATTACGCGGAGCGGG		35S, XVE, HSP
FAF1, FAF2	amiRFAF1/2	TCTCAGTGCACATCGCAACGC		35S
FAF3, FAF4	amiRFAF3/4	TCTCAGTGCACAATGCGACGC		35S
FAF2, FAF4	amiRFAF2/4-I	I: TTTCAACGAACTTTATCGGGG	At2g05830	35S, XVE, HSP
		II: TAATCAATCAAGATAGCGGGT		
	amiRFAF2/4-II	I: TTTCAACGAACTTTATCGGGG	At2g05830	35S, XVE, HSP
		II: TCAACGATTACGCGGAGCGGG		
	amiRFAF2/4-III	I: TTGAACTACAATTCGGCCGTC		35S, XVE, HSP
		II: TAATCAATCAAGATAGCGGGT		
	amiRFAF2/4-IV	I: TTGAACTACAATTCGGCCGTC		35S, XVE, HSP
		II: TCAACGATTACGCGGAGCGGG		
FAF4, FAF2	amiRFAF4/2-I	I: TAATCAATCAAGATAGCGGGT		35S, XVE, HSP
		II: TTTCAACGAACTTTATCGGGG	At2g05830	
	amiRFAF4/2-II	I: TAATCAATCAAGATAGCGGGT		35S, XVE, HSP
		II: TTGAACTACAATTCGGCCGTC		
	amiRFAF4/2-III	I: TCAACGATTACGCGGAGCGGG		35S, XVE, HSP
		II: TTTCAACGAACTTTATCGGGG	At2g05830	
	amiRFAF4/2-IV	I: TCAACGATTACGCGGAGCGGG		35S, XVE, HSP
		II: TTGAACTACAATTCGGCCGTC		

1) predicted targets according to WMD3, allowing for max. 5 mismatches, using TAIR9 genome annotation; 2) sequence of the amiRNA; 3) promoters used: 35S=CaMV-35S, XVE=estradiol inducible promoter (Zuo J., Niu Q.W., Chua N.H. (2000) Technical advance: an estrogen receptor-based transactivator XVE mediates highly inducible gene expression in transgenic plants. Plant J. 24(2): 265-273), HSP=heat-shock inducible promoter (AthHSP18.2; At5g59720); 4) indicates constructs in which two amiRNA foldback structures (both based on Ath-miR319 backbone) were cloned in tandem.

Table S5: Summary of *Arabidopsis thaliana FAF* tilling lines.

Gene	SNP[1]	Polymorphism	NASC[2]	CS[3]
FAF1	G>A	96C1	N85728	CS85728
	G>A	116C4	N86554	CS86554
	C>T	115G5	N87237	CS87237
	G>A	143G5	N90721	CS90721
	G>A	149C7	N91181	CS91181
	C>T	168E1	N91604	CS91604
	G>A	192F8	N94073	CS94073
FAF2	C>T	107D1	N86101	CS86101
	G>A	103G3	N86320	CS86320
	C>T	110G8	N86911	CS86911
	G>A	117B8	N87364	CS87364
	C>T	123H8	N87635	CS87635
	C>T	126F3	N87789	CS87789
	G>A	174A7	N91773	CS91773
	G>A	172C1	N91872	CS91872
FAF3	C>T	94B2	N85589	CS85589
	C>T	94B5	N85614	CS85614
	G>A	102G2	N86226	CS86226
	C>T	195B4	N92215	CS92215
	G>A	196H1	N92268	CS92268
	C>T	197B1	N92338	CS92338
	G>A	189G8	N93811	CS93811
	C>T	192G5	N94050	CS94050
FAF4	G>A	98C6	N85939	CS85939
	C>T	101A6	N86181	CS86181
	G>A	198G8	N92474	CS92474
	C>T	190F1	N93826	CS93826
	C>T	192H1	N94018	CS94018
	G>A	192C8	N94070	CS94070

1) SNP = single nucleotide polymorphism; 2) identification number of seed stock at NASC (The European Arabidopsis Stock Centre;); 3) identification number of seed stock at ABRC (Arabidopsis Biological Resource Center)

Table S6. Overview of oligonucleotides used in this study.

Gene	Name	Sequence (5′→3′)	strand
**Cloning *Arabidopsis thaliana FAF* ORFs[1]**			
At4g02810 (*FAF1*)	G-135	gatctgcagATGTCCATCGTTGTTGGCCAAGCT	sense
	G-136	atcggatccTTAAGTGGTCACCCAAAATTGCTG	antisense
At1g03170 (*FAF2*)	G-137	gatctgcagATGTCACTTGTTGTTTGTCAACCT	sense
	G-138	atcggatccTTAAGTAGCGACCCAAAACTGCTG	antisense
At5g19260 (*FAF3*)	G-139	gatctgcagATGGGAACTGTTGTGTATCAACAA	sense
	G-140	atcggatccTTAGGAAGTAGCAACGCAGAAAGA	antisense
	G-141	gatctgcagATGGCAACTGTTGTATATCAATCT	sense
At3g06020 (*FAF4*)	G-142	atcggatccTTAGGAAGTGGCAACACATAAAGA	antisense
**Cloning *Arabidopsis thaliana FAF* 2.5kb promoter fragments[1]**			
At4g02810 (*pFAF1*)	G-1142	gcgctgcagGTGCTCAAATAAATGCATAGTTGAAG	sense
	G-1143	cgcggatccTTTTTGTGT TGTGTTAAGAGAGC	antisense
At1g03170 (*pFAF2*)	G-1140	cgcgaattcTTATACTACACACGTTTTTAACTGGTC	sense
	G-1141	cgcggatccTTTTCTATGTTCTTTAGAAAAATAGC	antisense
At5g19260 (*pFAF3*)	G-1146	cgcgaattcACCGAATATTGAAACGTGTCTTGGGTAG	sense
	G-1147	gccggatccAGTTTAAAGATGATAGAAGAAAAACTG	antisense
At3g06020 (*pFAF4*)	G-1144	cgcgaattcTTATCGATATCTTAACAATTTTAAGAG	sense
	G-1145	gccggatccAAAGTTCAAAGATGAAGATTTCTTG	antisense
**Quantitative real-time RT-PCR[1]**			
FAF1	G-0662	ACGAGGAGGGCATTGTGGGGAATAATG	sense
	G-0663	TTAAGTGGTCACCCAAAATTGCTGCTG	antisense
FAF2	G-0664	CTTCGTCTCATTTTAACGTCGGAAAGT	sense
	G-0665	GGTTTAGGCTCACGACCATTCTCCTTG	antisense
FAF3	G-0668	GGGAAACAACAACGGAGACAAGAACGT	sense
	G-0669	GAGACGACCATTGCTACGGTCAGCTTG	antisense
FAF4	G-0666	TTCCACCTCCTTTGACAAGTATGATAG	sense
	G-0667	CTTCGTTATCTCTCACGGTCTCGATTG	antisense
β-Glucuronidase	G-1563	CTGCATCAGCCGATTATCATCACC	sense
	G-1564	ACCGAAGTTCATGCCAGTCCAGCG	antisense
β-Tubulin	N-78	GAGCCTTACAACGCTACTCTGTCTGTC	sense
	N-79	ACACCAGACATAGTAGCAGAAATCAAG	antisense

1) Sequences matching the *Arabidopsis* ORF are given in capital letters

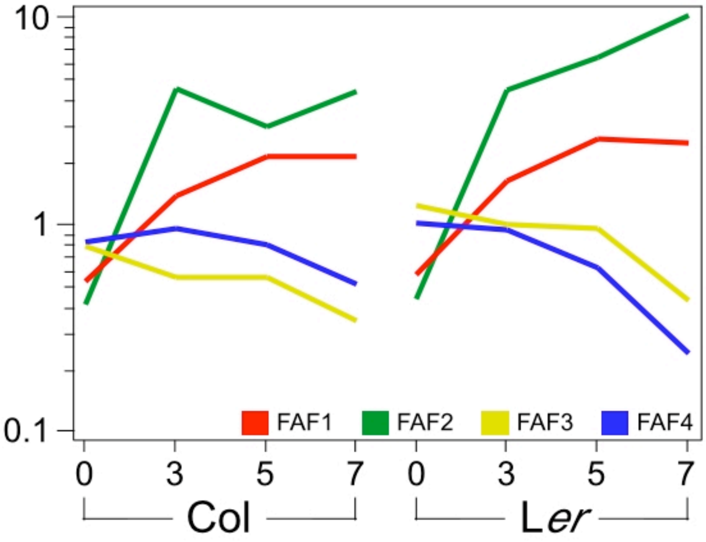

Figure S1. Expression profiles of *FAF* genes in response to long day (LD). Plants were grown under short day conditions for 30 days before plants were shifted to LD. Gene expression was monitored by Affymetrix microarrays 0, 3, 5, and 7 days after the shift to LD [1].

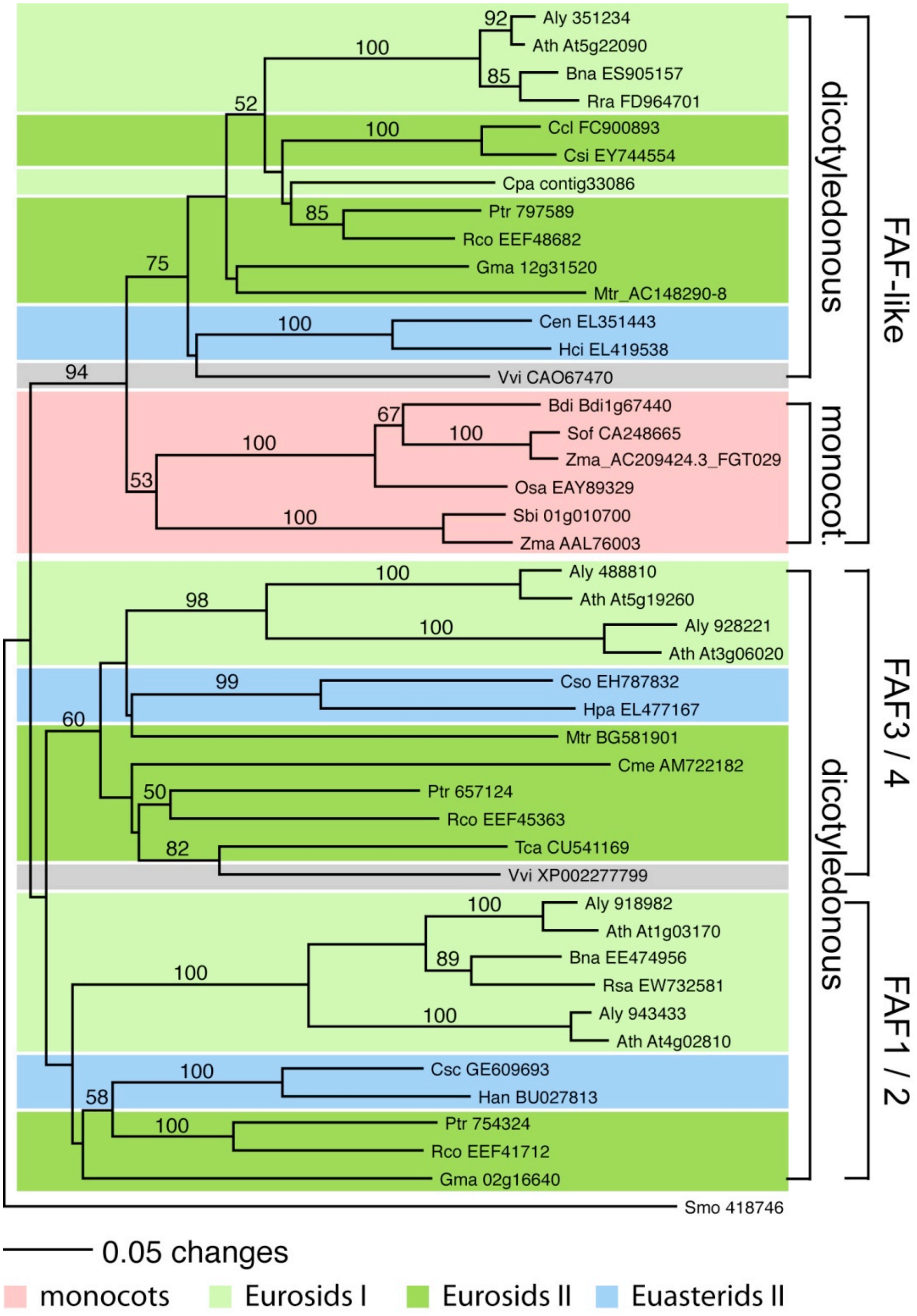

Figure S2. Phylogenetic analysis of the plant-specific FAF protein family using Neighbor-Joining method. Sequences from the following species were used to construct the tree: *Arabidopsis lyrata* (Aly), *Arabidopsis thaliana* (Ath), *Brachypodium distachyon* (Bdi), *Brassica napus* (Bna), *Carica papaya* (Cpa), *Centaurea solstitialis* (Cso), *Cichorium endivia* (Cen), *Citrus clementina* (Cle), *Citrus sinensis* (Csi), *Cucumis melo subsp. Melo* (Cme), *Cynara scolymus* (Csc), *Glycine max* (Gma), *Helianthus annuus* (Han), *Helianthus ciliaris* (Hci), *Helianthus paradoxus* (Hpa), Medicago

truncatula (Mtr), *Oryza sativa* (Osa), *Populus trichocarpa* (Ptr), Raph*anaus raphanistrum subsp. Maritimus* (Rra), *Raphanus sativus* (Rsa), *Ricinus communis* (Rco), *Saccharum officinarum* (Sof), *Selaginella moellendorffii* (Smo), *Sorghum bicolor* (Sbi), *Theobroma cacao* (Tca), *Vitis vinifera* (Vvi) and *Zea mays* (Zma). Numbers in the protein names refer to Genbank and Phytozome identifiers. Topological robustness was assessed by bootstrap analysis with 1000 replicates using simple taxon addition [2].

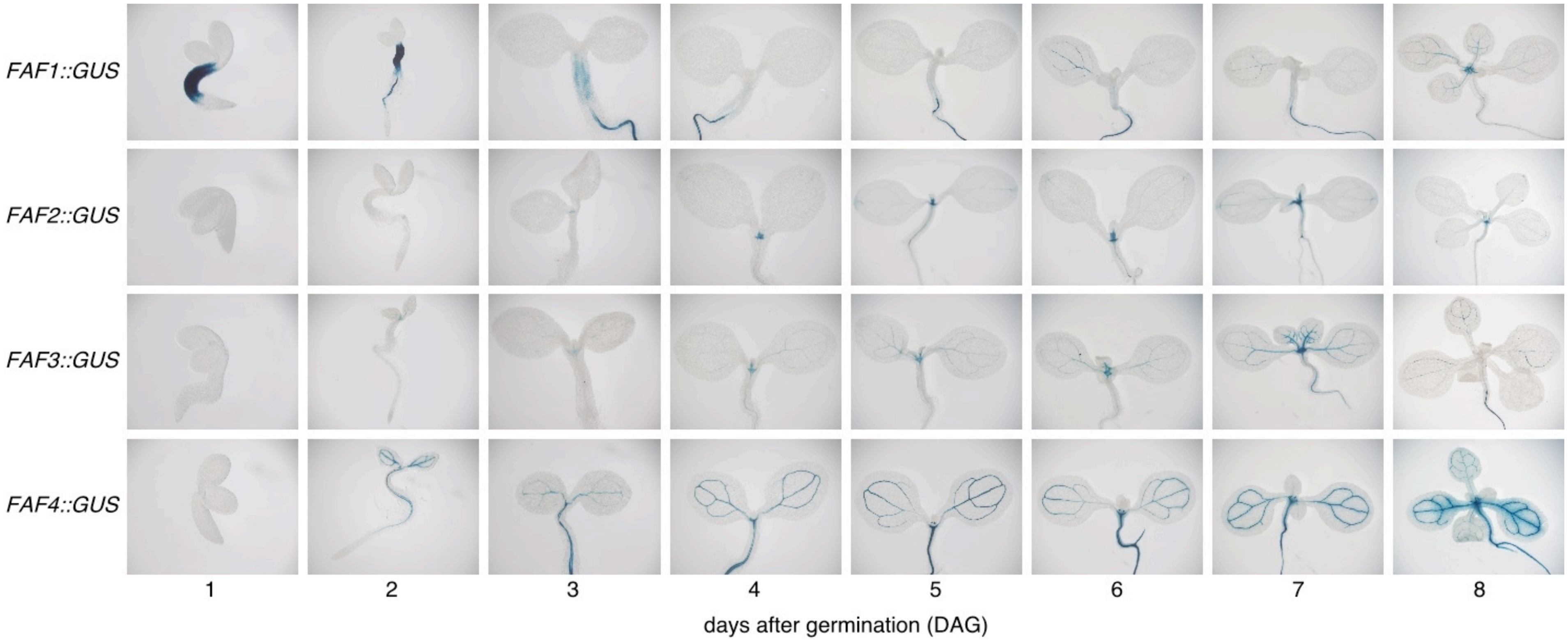

Figure S3. Changes in *GUS* expression in FAF reporter lines. The activity of *FAF1::GUS* (top row), *FAF2::GUS* (2nd row), *FAF3::GUS* (3rd row), *FAF4::GUS* (bottom row) throughout the first 8 days after germination (DAG) is shown.

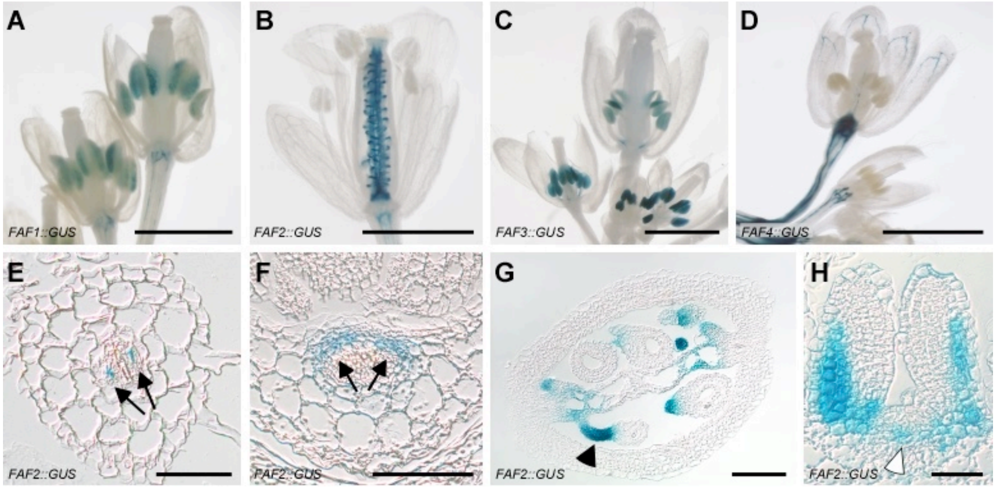

Figure S4. *FAF::GUS* reporter gene activity in the meristem and reproductive organs.

Expression of *FAF1* (**A**) and *FAF3* (**C**) was mostly limited to anthers, while *FAF2* was found in the funiculus (**B**, **G** arrowhead). *FAF4* expression was restricted to the pedicel and inflorescence stem (**D**). (**E**, **F**) Vascular expression of *FAF2* was strongest in the phloem of roots (**E** arrows) and leaves (**F** arrows). (**H**) *FAF2* activity in the centre of the meristem was also observed in RNA *in situ* hybridization (Fig. 3M arrowhead).

Supplementary References:

1. Schmid M, Uhlenhaut NH, Godard F, Demar M, Bressan R, Weigel D, Lohmann JU: **Dissection of floral induction pathways using global expression analysis**. *Development* 2003, **130**(24):6001-6012.
2. Felsenstein J: **Confidence limits on phylogenies: an approach using the bootstrap**. *Evolution* 1985, **39**(4):783-791.
